# Evaluation of the Improvement Effect of Whey Protein Poly-Peptides on Quality Characteristics of Repeated Freeze–Thawed Spanish Mackerel Surimi Balls

**DOI:** 10.3390/foods13030403

**Published:** 2024-01-26

**Authors:** Xiaowen Zhang, Shaojing Zhong, Lingru Kong, Xiaohan Wang, Juan Yu, Xinyan Peng

**Affiliations:** 1 College of Life Sciences, Yantai University, Yantai 264005, China; zxw19861108608@163.com (X.Z.); konglr2226@163.com (L.K.); 15966907501@163.com (X.W.); yujuan14615@163.com (J.Y.); 2 Yantai New Era Health Industry Daily Chemical Co., Ltd., Yantai 264005, China; 17853510711@163.com

**Keywords:** surimi balls, whey polypeptide, water retention, antioxidant, sensory evaluation

## Abstract

This investigation aimed to assess the effects of whey protein hydrolysate (WPH) on the oxidative stability of protein and the ability of *Scomberomorus niphoniu* surimi balls to retain water after repeated freeze–thaw (F–T) cycles. Ten percent natural whey peptides (NWP), 5% WPH, 10% WPH, 15% WPH, 0.02% butyl hydroxyl anisole (BHA), and a control group that did not receive any treatment were the six groups that were employed in the experiment. The cooking loss, water retention, total sulfhydryl content, and carbonyl content of each group were all measured. Notably, it was found that the surimi balls’ capacity to hold onto water and fend off oxidation was enhanced in a dose-dependent manner by the addition of WPH. Furthermore, the results showed that the 15% WPH added to the surimi balls effectively decreased protein oxidation in the F–T cycles and ameliorated the texture deterioration of surimi balls induced by repeated F–T, laying a theoretical foundation for the industrial application of WPH in surimi products.

## 1. Introduction

Products made from surimi balls are gel-based meals with various flavors made by selecting, crushing, drying, and rinsing raw fish [1]. Preservatives and flavoring agents are also added [2]. Freezing is one of the most common methods for preserving surimi balls [1]. However, the texture and gel qualities of repeatedly frozen and thawed surimi balls will be severely deteriorated by changes in ambient temperature during shipping, storage, sales, and other links [3,4]. Numerous investigations have shown that ice crystals’ production, melting, and regeneration cause harm brought on by the freeze–thaw (F–T) cycle. Consequently, the expansion and dispersion of ice crystals cause the structural distortion and rupture of muscle cells, puncturing myofiber cells and inflicting mechanical damage to cell membranes and tissue structures [5,6]. Furthermore, the pores that ice crystals leave behind can hasten the development of undesirable metabolic reactions in surimi balls, such as lipid oxidation and protein denaturation [7]. Additionally, the integrity of the muscles in the surimi balls gradually deteriorates with an increase in the number of F–T cycles, leading to significant differences in the variables that impact the commodity value, including color, gel performance, and water retention [8,9]. The distribution, shape, orientation, and particle size of the meat ice crystals will alter due to the repeated F–T process according to a study by Chen et al. [10]. Further, the muscle’s original myofibril spacing altered the water molecules that were lost from the muscle’s original space, and the formation of ice crystals reduced the muscle’s ability to hold onto water [11].

A growing body of research has demonstrated in the last several years that protein oxidative denaturation is one of the main factors contributing to surimi ball quality degradation and sap loss during the F–T process [12,13,14]. Because surimi is prone to protein oxidation, interactions with lipid oxidation inter-mediates may form carbon groups, which can alter the physical and functional characteristics of proteins and cause the loss of solubility and fragment aggregation [15]. The pro-oxidants found in fish, such as iron and hemoglobin [16], easily combine with other materials before, during, and following enzymatic hydrolysis to generate negative oxidation products [17]. Several variables affect how much water beef tissues retain, and the oxidative denaturation of meat proteins may be the reason for even a slight change in ice crystal morphologies [8]. According to previous research [18], oxidizing myofibrillar proteins, destroying the tertiary structure, exposing the hydrophobic domain, and changing the surface charge and hydrogen bonding distribution all result in modifications to the protein conformation. Chaijan et al. [19] found that the initial spacing of myofibrils altered when ice crystals developed, which led to the loss of water molecules stored in the original space in the muscle and a decrease in the muscle’s ability to retain water. According to the study of An et al. [20], pork’s thiobarbituric acid value and carbonyl group content are dramatically lowered by potato hydrolyzed polypeptide, which can significantly block the oxidation of fat and protein. Li et al. [21] also found that adding whey polypeptides during freezing effectively inhibited the oxidation of surimi’s myofibrillar protein. However, the impact of whey peptide hydrolysate on the quality of repeatedly freeze–thawed surimi balls has not been thoroughly studied.

Research has demonstrated that whey polypeptides possess a potent antioxidant potential [11,19,22]. As a result of their elevated biological activity as antioxidants, scholars have recently focused extensively on whey polypeptides derived from whey protein [21,22]. After being frozen and then thawed, the gel has an excellent network structure, is tight and homogeneous, and is better able to hold onto the texture of the protein gel [21]. This experiment compared butyl hydroxy anisole and natural whey protein with varying amounts of whey polypeptide hydrolysate. Since there is not much research on the topic, a blank control was set up to examine how much natural whey polypeptides should be added while repeatedly freezing and thawing surimi balls. This research will create the theoretical foundation for the preservation and deep processing of surimi ball products in China, in addition to offering technological assistance for the growth of the surimi ball processing sector. This research will further enhance the quality of China’s aquaculture products, assist the industry that produces surimi balls, and progressively increase the country’s exports of surimi ball goods.

## 2. Materials and Methods

### 2.1. Chemicals and Materials

Fresh Spanish mackerel (*Scomberomorous niphonius*) was purchased from Zhenhua Supermarket in Yantai, China. Natural whey protein (NWP, 95%) was bought from Davisco Foods International, Inc. in Minneapolis, MN, USA. 1,1,3,3-tetraethoxypropane, trichloroacetic acid (TCA), ethylene diamine tetra acetic acid (EDTA), and butylhydroxyanisole (BHA) were purchased from Sigma Corporation, St. Louis, MO, USA. Tris-HCl and Sodium dodecyl sulfate (SDS) were purchased from Beijing Dingguo Chansheng Bio-Technology Co., Ltd. in Beijing, China. Additionally, all the other chemicals (NaCl, HCl, ethyl acetate, ethanol) used in the experiment were of analytical grade and bought from Sinopharm Chemical Reagent Co., Ltd. in Shanghai, China.

### 2.2. Preparation of Surimi Balls

Following deboning, the fresh fish (weight of 450 ± 30 g) was randomly divided into six groups and minced using a meat grinder (SK-088, SOKANY, Berlin, Germany). The control group consisted of surimi ball samples that had not been treated with additives. In this order, 10% NWP, 5% WPH, 10% WPH, 15% WPH, and 0.02% BHA were added to the remaining five groups. Next, we added 2.5% NaCl to the minced surimi combination in each group. All groups were made into 30 mm diameter surimi balls. Each group was measured three times. The temperature was kept at about 4 °C for the surimi ball production procedure. The samples were frozen for five days at −18 °C. They were then thawed at 4 °C for 12 h, allowing the core temperature to approach 0~2 °C and end the freeze–thaw cycle. We repeated F–T cycles 0, 1, 3, 5, and 7 according to the previously described protocol [17].

### 2.3. Water Holding Capacity (WHC) of Surimi Balls

The WHC of the surimi balls was performed according to the method of Fan et al. [23], with minor modifications. The samples of surimi balls were precisely weighed (m_1_) after being allowed to acclimate to room temperature (4 °C). The surimi balls were weighed again and recorded as m_2_ before being wrapped in two filter papers and centrifuged (Avanti J-E, Beckman Coulter, Pasadena, CA, USA) at 5630× *g* for 10 min at 4 °C. The WHC was calculated by the following formula:

WHC (%) = m_2_/m_1_ × 100 (1)

### 2.4. Elasticity of Surimi Balls

The elasticity of the surimi balls was tested during the F–T cycles using the approach of Cao et al. [24], with modifications. To assess the elasticity of surimi balls using the texture analyzer (TA.XT Plus, SMS, Stable Micro Systems Ltd., Godalming, UK), we used a cylindrical probe (P/36R) with a compression distance of 15 mm, a deformation percentage of 75%, and a trigger force of 10 g. Each sample group was measured three times in parallel before calculating the average value.

### 2.5. Chewiness of Surimi Balls

The method described by Jiang et al. [25], with minor alterations, was used to measure chewiness. A cylindrical P/0.25S probe and a TA-XT Plus texture analyzer (TA.XT Plus, SMS, Stable Micro Systems Ltd., Godalming, UK) were used to quantify and analyze the chewiness characteristics at a constant test speed of 1.0 mm/s, a piercing distance of 15 mm, and a trigger value of 5 g. Three parallel measurements were made for each sample group, from which the average value was derived. Notably, chewiness is the end product of elasticity and adhesiveness. The degree to which the surimi balls “spring back” after the first puncture among them signifies their elastic nature; on the other hand, the degree to which they withstand deformation from the puncture during the second puncture signifies their adhesive nature.

### 2.6. Hardness of Surimi Balls

According to the previous study by Zhang et al. [26], the hardness of surimi balls was determined. The surimi ball byproduct was utilized to make three-centimeter-long cylinders. Moreover, the hardness of the surimi balls product was measured using a texture analyzer equipped with a probe (P/36R). The pre-test, post-test, and test speeds were set at 2 mm/s.

### 2.7. Cooking Loss in Surimi Balls

The technique of Kim et al., with minor modifications, was used to detect the mackerel surimi steaming water loss [27]. The fresh surimi balls each weighed 5 g, and the weight M was noted. After 20 min of steam-cooking, the weight M_0_ was recorded, and the items were cooled at 4 °C for a full 24 h. To ascertain the cooking loss, the following formula was employed:

(2) $$\mathrm{Cooking}\;\mathrm{loss}=\frac{M-M_{0}}{M}\times 100\%$$

### 2.8. Total Sulfhydryl Content

The total sulfhydryl content of the surimi was determined via the modification of the procedures outlined by Yu et al. [28] and Walayat et al. [29]. One gram of surimi was homogenized with 9 mL of 0.8% saline. A mixture was prepared with 0.5 mL of homogenate solution and 4.5 mL of buffer solution (consisting of 0.2 mol/L Tris-HCl, 1% SDS, 10 mmol/L EDTA, and 8 mol/L urea, at pH 7.0). Next, 4 mg/mL of the above mixture was incorporated into Ellman’s reagent (0.5 mL) and Tris-HCl (0.2 M, pH 8.0), which was thoroughly agitated and then incubated at 45 °C for 20 min. Afterward, the absorbance of the supernatant was measured at 412 nm to ascertain the total sulfhydryl content, employing a molar extinction coefficient of 13,600 mol/L^−1^ cm^−1^. This approach aimed to reduce the similarities with the previously published research.

### 2.9. Carbonyl Content of Surimi Balls

The carbonyl content of surimi balls was measured using the method described by Wang et al. [30]. First, 1 g of surimi balls was homogenized in 0.2 M NaCl at pH 6.5, and the mixture was centrifuged for 15 min at 13,000× *g* at 4 °C. Then, 0.4 mL of the supernatant was extracted again to determine the amount of carbonyl compounds. In one case, the protein was precipitated using 1 mL of 2,4-dinitrobenzene solution combined with 2 M HCl, while the control group received 0.2 mL of 2 M HCl. Next, we added 1 milliliter of a solution containing 40% TCA and let it incubate for 20 min. After centrifuging the sample at 4 °C for 10 min at 13,000× *g*, we rinsed the precipitated proteins with ethyl acetate/ethanol (*V*/*V* = 1:1) solution, removed the unreacted solvent, and continued the washing process above until the supernatant was colorless. After dissolving the sample pellet in 3.5 mL of 6 M guanidine hydrochloride, the absorbance was measured at 370 nm. In order to calculate the carbonyl concentration, the protein nmol/mg absorbent coefficient was used.

### 2.10. Sensory Evaluation

The sensory evaluation of fish balls adhered to the methodology of Qiu et al. [31], with a few minor modifications. This was conducted in the 25 °C sensory analysis room by ten exceptionally knowledgeable and experienced individuals (five men and five women). Panel members were asked to refrain from eating and smoking for one hour before the trial. After thawing, the surimi balls were cooked to 90 °C and cooled to 25 °C. A random three-digit number was used to encrypt each sample. We used a 10-point rating system to evaluate the sensory qualities of the surimi balls. Before every test, participants were instructed to rinse their mouths with pure water to prevent confounding. Following the experiment’s F–T cycles, sensory analysis was carried out three times, and the average score for each sample was determined.

### 2.11. Statistical Analysis

The data were processed using an analysis of variance (ANOVA) and a general linear model program. Statistics 8.1 (Analytical Software, St Paul, MN, USA) was used for all statistical analyses. Tukey multiple comparisons were employed to establish the effect’s significance (*p* < 0.05). Using SPSS Statistics 16.0, the data were examined for variance homogeneity and normality. All data are expressed as mean ± standard error (SE). The illustration was produced utilizing Sigmaplot 12.0 software.

## 3. Results and Discussion

### 3.1. WHC of Surimi Balls

**Figure 1:**
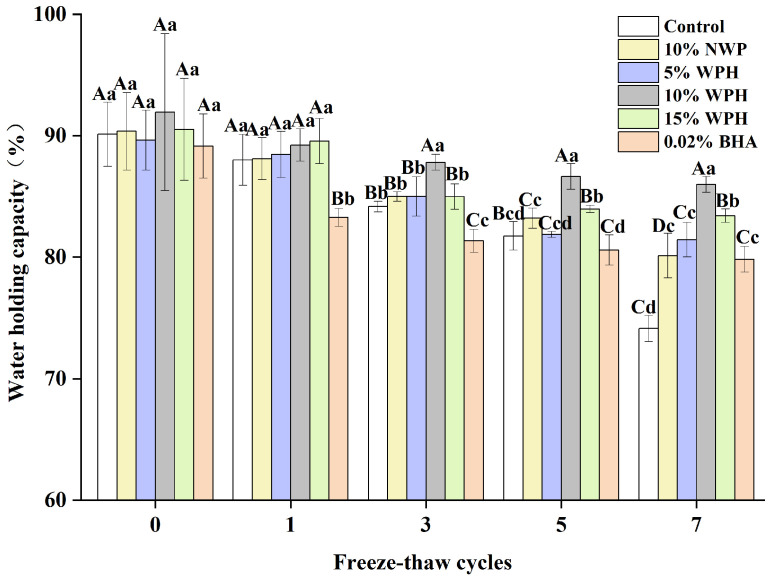
Effects of WPH on the water-holding capability of surimi balls during repeated F–T cycles. Uppercase letters (A–D) indicate significant differences between distinct cycles. Different lowercase letters (a–d) indicate significant differences between treatments. Control: without additives in the sample; NWP: natural whey protein; WPH: whey protein hydrolysates; BHA: butyl hydroxy anisole.

The water-holding capacity of surimi balls is critical in determining the quality of meat products and how well the balls hold water [32]. The samples’ WHC varies as the number of F–T cycles rises, as shown in Figure 1. The WHC of the frozen samples dropped from an initial range of 89.14–91.94% to 74.12–85.99% after seven F–T cycles. As the frequency of F–T cycles grew, the WHC of surimi balls progressively decreased, which was in line with research showing that F–T cycles cause a decrease in the fish ball WHC [33]. Protein deterioration and myofibril disintegration may alter the cell structure, which could account for this occurrence [34]. Compared to the 10% NWP and 5% WPH additions, the WHC of surimi balls did not significantly improve with 0.02% BHA and 15% WPH after three and five F–T cycles. When 10% WPH was added, the WHC of surimi balls was significantly (*p* < 0.05) higher than the control. After seven F–T cycles, the 10% WPH addition’s WHC reached 85.99%, outperforming the results of the 0.02% BHA and the 15% WPH additions. This could be because surimi balls have a relatively high WHC. Afterall, the proteolytic peptides inhibit changes in the spatial structure of myofibrils and limit muscle tissue water loss during F–T cycles [22,35].

### 3.2. Elasticity of Surimi Balls

**Figure 2:**
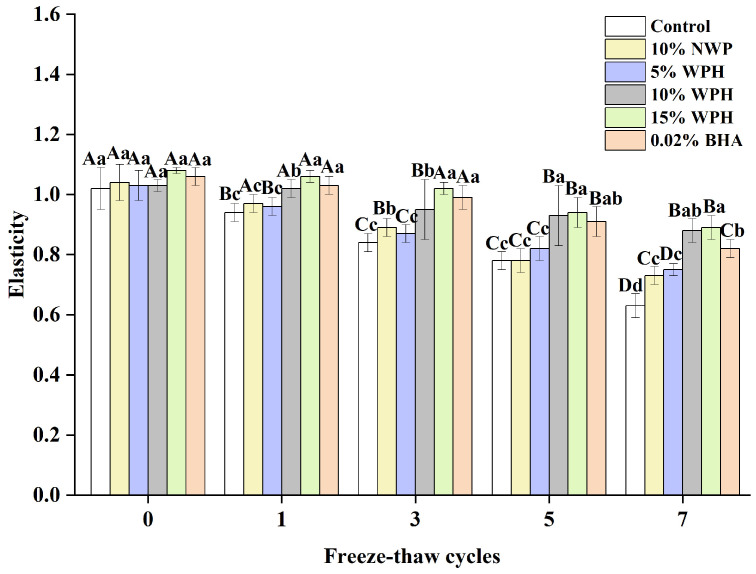
Effect of WPH on the elasticity of surimi balls during repeated F–T cycles. Uppercase letters (A–D) indicate significant differences between distinct cycles. Different lowercase letters (a–d) indicate significant differences between treatments. Control: without additives in the sample; NWP: natural whey protein; WPH: whey protein hydrolysates; BHA: butyl hydroxy anisole.

The consumer approval of products containing frozen surimi balls is significantly influenced by elasticity [36]. Figure 2 shows how the F–T cycle treatment affects the elasticity of surimi balls. The surimi balls’ elasticity quickly decreased as the F–T cycle period lengthened. Although there were observable changes in the surimi balls during the F–T cycles, the elasticity did not differ significantly after the 0th cycle (*p* > 0.05). It was also observed by Wang et al. [37] that surimi’s flexibility reduced with F–T cycles. The flexibility of the surimi balls significantly decreased with the increase in the number of F–T cycles, which could be explained by the longer-term compression of ice crystals, causing the greater deformation of muscle fibers due to shrinkage and dehydration [36,38]. The elasticity of the surimi balls in the blank control group decreased from 1.02 to 0.78 and finally to 0.63 after seven F–T cycles. The elasticity of the surimi balls decreased by 0.26 in the 10% WPH addition group and only by 0.19 in the 15% WPH group, demonstrating a slower change in the WPH groups. This difference suggests that the WPH groups were more significant as the number of F–T cycles rose. When compared to the 0.02% BHA group, the 10% WPH and 15% WPH groups showed a similar but slower declining trend, suggesting that the WPH embedded in the myofibrillar protein gel network preserved the reticular spatial structure of proteins and enhanced the surimi balls’ quality [39]. In particular, the samples with 15% WPH added had the least drop, and those with 10% WPH came in second.

### 3.3. Chewiness of Surimi Balls

**Figure 3:**
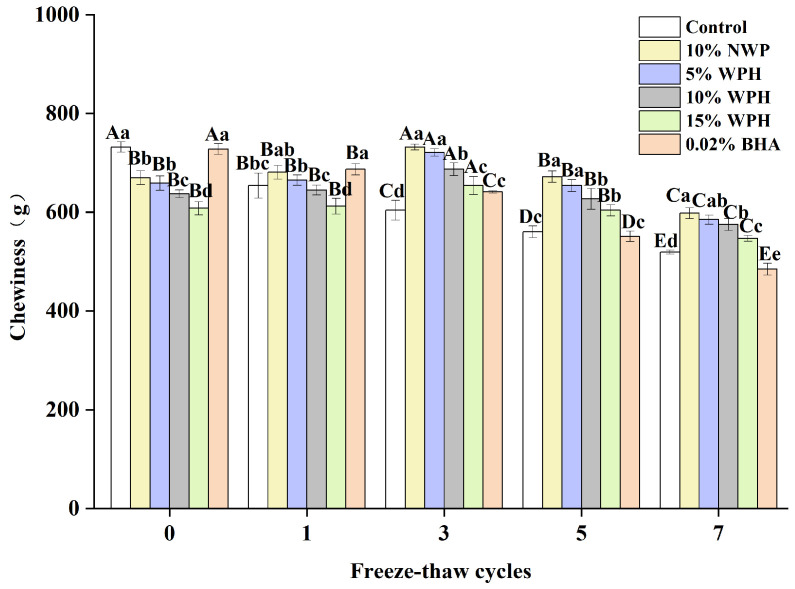
Effects of WPH on chewiness of surimi balls during repeated F–T cycles. Uppercase letters (A–E) indicate significant differences between distinct cycles. Different lowercase letters (a–e) indicate significant differences between treatments. Control: without additives in the sample; NWP: natural whey protein; WPH: whey protein hydrolysates; BHA: butyl hydroxy anisole.

Chewiness affects customer satisfaction and preference and is an important indicator of fresh fish [22]. The chewiness of surimi balls after seven F–T cycles is shown in Figure 3. It is evident that the chewiness of the WPH and NWP groups increased at first, then decreased as the F–T periods increased, peaking at the third F–T cycle (654–732 g). This could be connected to physical changes in the protein extraction capability, solubility, and viscosity during frozen storage [40]. According to our research, the chewiness of the fresh sample varied from 608 to 732 g. Conversely, following seven F–T cycles, the chewiness of surimi balls decreased to 484–598 g. This could be attributable to mechanical muscle cell disintegration and protein denaturation [41]. The chewiness of the 10% and 15% WPH groups did not change significantly after zero, one, and five F–T cycles (*p* > 0.05). In contrast, at the seventh F–T cycle, the 15% WPH and 0.02% BHA samples had less chewiness than the 5% WPH and 10% WPH groups (*p* < 0.05). After determining that the WPH addition reduces protein denaturation and the mechanical disruption of muscle cells, we concluded that the 15% WPH addition may successfully prevent the texture deterioration of F–T surimi balls and preserve their quality.

### 3.4. Hardness of Surimi Balls

**Figure 4:**
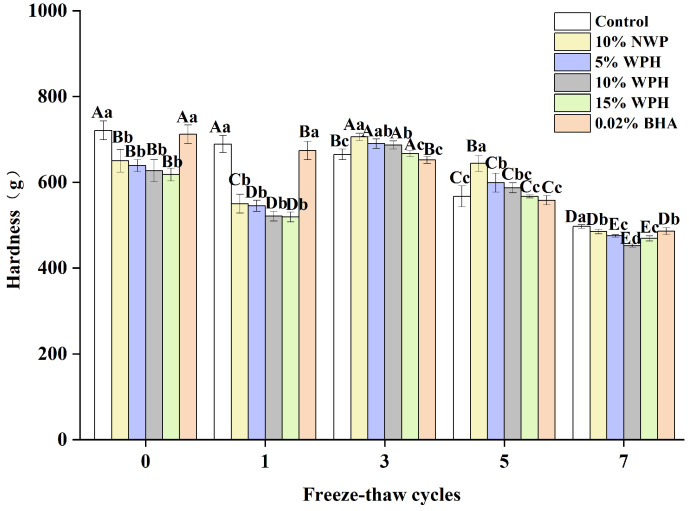
Effect of WPH on the hardness of surimi balls during repeated F–T cycles. Uppercase letters (A–E) indicate significant differences between distinct cycles. Different lowercase letters (a–d) indicate significant differences between treatments. Control: without additives in the sample; NWP: natural whey protein; WPH: whey protein hydrolysates; BHA: butyl hydroxy anisole.

The hardness of surimi balls after F–T cycles is a proxy for their degree of denaturation [36]. Figure 4 shows the surimi balls’ hardness following seven F–T cycles. The sample’s initial hardness was still between 618 and 721 g, and the groups that received 0.02% BHA and the blank group had significantly higher hardness than the other four groups (*p* < 0.05). The NWP and WPH groups’ surimi balls’ hardness tended to decline, then rise and fall. The surimi balls’ hardness reached their maximum value after three freeze–thaw cycles. The results of Walayat et al. [42] regarding the gel properties of silver carp surimi balls are consistent with this pattern. Larger particle sizes in NWP and WPH may cause this occurrence, since they exert more pressure on the protein matrix and possibly increase its hardness [26]. Following seven F–T cycles, the hardness of the control, 10% NWP, 5% WPH, 10% WPH, 15% WPH, and 0.02% BHA samples dropped by 31%, 25.4%, 25.8%, 27.8%, 24.1%, and 37.1%, respectively. An increase in hardness is thought to have resulted from the WPH binding to proteins in the network spaces [19].

### 3.5. Cooking Loss of Surimi Balls

**Figure 5:**
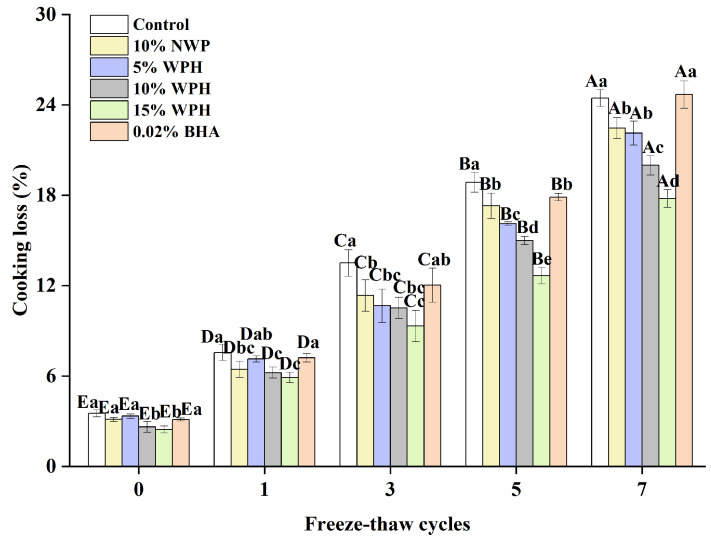
Effect of WPH on the cooking loss of surimi balls during repeated F–T cycles. Uppercase letters (A–E) indicate significant differences between distinct cycles. Different lowercase letters (a–e) indicate significant differences between treatments. Control: without additives in the sample; NWP: natural whey protein; WPH: whey protein hydrolysates; BHA: butyl hydroxy anisole.

The presence of non-surimi ingredients is identified as the primary cause of the cooking loss in surimi products, which is a significant indicator of the type of cooking loss that surimi displays [43]. The proteins in surimi are lost during cooking because myofibrillar proteins shrink at high temperatures, which decreases the proteins’ capacity to bind with water [44]. The impact of various component additions on surimi’s cooking loss during the freeze–thaw cycle is depicted in Figure 5. No discernible difference between the groups was evident (*p* > 0.05), and the cooking loss was less than 5% when the freeze–thaw cycle was not in effect. After a single F–T cycle, the cooking loss rose; yet, the inclusion of the WPH group increased more gradually. Following three F–T cycles, the rate of increase was greatly accelerated and increased with the number of F–T cycles. There was a significant difference (*p* < 0.05) in cooking losses with the number of freezing and thawing cycles. The addition of various additive groups could effectively inhibit the rise in the cooking loss; the effect of the 15% WPH addition group was the most significant, with a rise of only 15.33%. The maximum was reached after seven freeze–thaw cycles. Among the experimental groups, the highest cooking loss was 24.68% in the 0.02% BHA group, which was similar to that of the control group. This might be a result of WPH’s inherent ability to retain water; adding it to surimi will increase the protein–water interaction and increase the amount of water trapped in the gel network [45]. Consequently, it can be said that the addition of 15% WPH has the greatest inhibitory effect on cooking loss and has a good impact on the preservation of water loss in surimi.

### 3.6. Total Sulfhydryl Content

**Figure 6:**
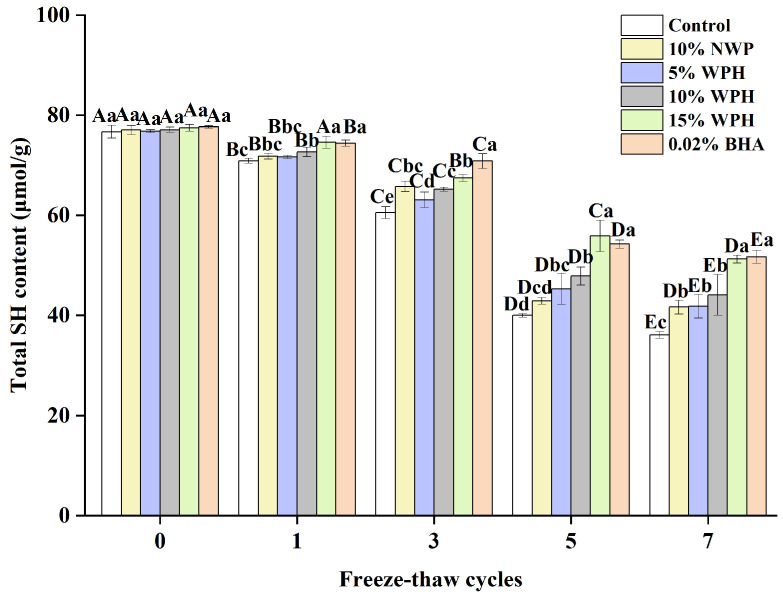
Effect of WPH on total sulfhydryl content of surimi balls during repeated F–T cycles. Uppercase letters (A–E) indicate significant differences between distinct cycles. Different lowercase letters (a–e) indicate significant differences between treatments. Control: without additives in the sample; NWP: natural whey protein; WPH: whey protein hydrolysates; BHA: butyl hydroxy anisole.

The total sulfhydryl content on the side chain of the protein is the most active functional group and readily forms disulfide bonds via dehydrogenation, which oxidizes the proteins [46,47]. Figure 6 depicts the change in the total sulfhydryl content of different dosages of surimi balls during the F–T cycles. The total sulfhydryl content of the surimi balls reduced significantly as the number of F–T cycles increased (*p* < 0.05). This is comparable to the research conducted by Liu et al. [48], which found that a decrease in the water-holding capacity was caused by a significant reduction in the total sulfhydryl content of all MP samples when the freezing period was increased. There was no discernible variation in the total sulfhydryl content among the six groups of surimi balls at the 0th freeze–thaw cycle (*p* > 0.05). As the number of freeze–thaw cycles rose, the total sulfhydryl content of proteins susceptible to the F–T cycle decreased. This phenomenon may be related to the sulfhydryl group’s ability to form intra- or intermolecular disulfide linkages [49]. Another theory was that freezing caused ice crystals to develop, which compromised the cell integrity and encouraged the conversion of sulfhydryl linkages to disulfides, which lowered the concentration of sulfhydryl [46]. The total sulfhydryl content of the blank group, 10% NWP, 5% WPH, 10% WPH, 15% WPH, and 0.02% BHA group reduced by 52.4%, 45.9%, 45.6%, 42.8%, 33.9%, and 33.4%, respectively, after seven freeze–thaw cycles. It also declined following the freeze–thaw cycle, with an increase in the WPH addition; this might be due to persistent adducts forming between the sulfhydryl groups and WPH during the F–T cycle [48]. These adducts can enhance the creation of disulfide connections between proteins, thereby preventing the oxidation of sulfhydryl groups. Consequently, a reduction in the total sulfhydryl content of surimi was noted following seven F–T cycles. After one, three, five, and seven cycles, the total sulfhydryl content of the 15% WPH group and the 0.02% BHA group was significantly higher than that of the blank group, the 10% NWP, 5% WPH, and 10% WPH group; however, there were no significant differences between the groups of 10% NWP, 5% WPH, 10% WPH, and 10% WPH (*p* > 0.05). Thus, we concluded that the 15% WPH addition had the most significant effect on inhibiting sulfhydryl oxidation.

### 3.7. Carbonyl Content of Surimi Balls

**Figure 7:**
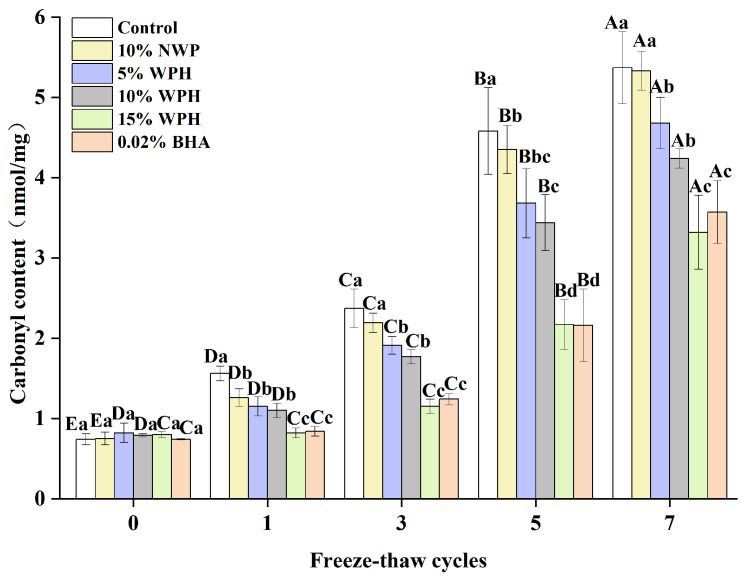
Effect of WPH on carbonyl content of surimi balls during repeated F–T cycles. Uppercase letters (A–E) indicate significant differences between distinct cycles. Different lowercase letters (a–d) indicate significant differences between treatments. Control: without additives in the sample; NWP: natural whey protein; WPH: whey protein hydrolysates; BHA: butyl hydroxy anisole.

The degree of protein oxidation can be determined by measuring changes in the carbonyl concentration, where a higher carbonyl content indicates a higher degree of oxidation [50]. The carbonyl content of surimi balls is shown in Figure 7 following seven F–T cycles. Initially, the carbonyl content of the six groups of surimi balls ranged from 0.74 to 0.82 nmol/mg. As the number of F–T cycles increased, the carbonyl content of the additive groups increased steadily, but no significant difference was seen during the 0th cycle (*p* > 0.05). Liu et al. [51] reported that the carbonyl level in bighead carp kept in frozen storage increased from 2.45 nmol/mg to 7.67 nmol/mg following seven F–T cycles. This impact could be explained by the tendency of the ice crystals created during the F–T cycles to pierce the gel tissue, accelerating the oxidation of proteins by stimulating the oxidation of fat and releasing more free radicals [52]. Sousa et al. [53] also showed that the synthesis of carbonyls is linked to the conformational changes of myofibrillar proteins, causing protein fragmentation and aggregation. After one, three, five, and seven F–T cycles, the carbonyl contents of the surimi balls in the 15% WPH and 0.02% BHA groups were equally effective, and both were considerably lower than that of the blank control group, 10% NWP, 5% WPH, and 10% WPH groups (*p* < 0.05). Following seven F–T cycles, the carbonyl content of surimi balls in the 15% WPH group climbed by 315%; this is compared to a 626% increase in the blank control group. Meanwhile, the carbonyl content of the 15% WPH addition was comparable to that of the 0.02% BHA group. The results showed that WPH had a specific inhibitory influence on the growth in the protein carbonyl content over repeated F–T cycles. Furthermore, the addition of 15% WPH had the highest inhibiting effect on the increase in the carbonyl content during numerous freeze–thaw processes.

### 3.8. Sensory Evaluation

**Figure 8:**
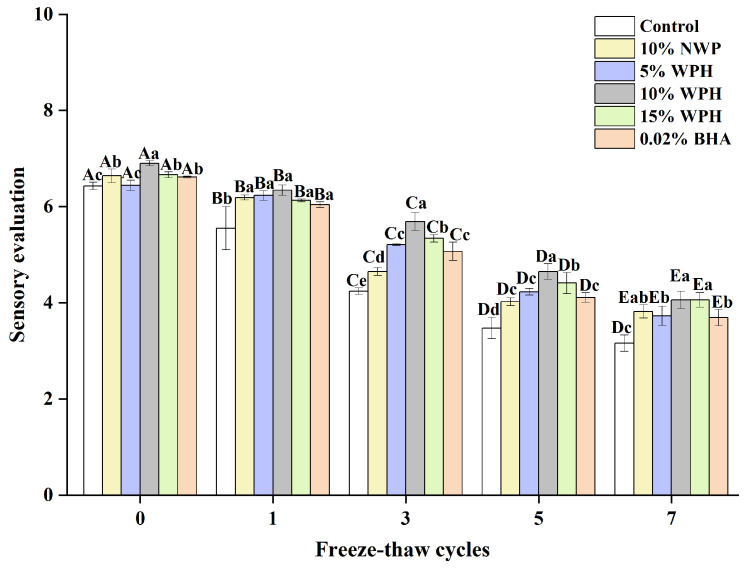
Effect of WPH on overall sensory evaluation of surimi balls during repeated F–T cycles. Uppercase letters (A–E) indicate significant differences between distinct cycles. Different lowercase letters (a–e) indicate significant differences between treatments. Control: without any additives in the sample; NWP: natural whey protein; WPH: whey protein hydrolysates; BHA: butyl hydroxy anisole.

The ability of WPH samples to stabilize emulsions, hold water and fat, reduce lipid oxidation, and bind/fill the protein gel network may be related to the slight changes in the sensory preference observed during F–T cycles [35]. Sensory evaluation can reveal fundamental differences in properties between surimi ball samples [54,55]. Figure 8 illustrates how various additions affect the overall sensory qualities of fish balls. After seven freeze–thaw cycles, the surimi balls in each group’s total sensory evaluation decreased. The surimi balls were sensorially rated significantly lower (*p* < 0.05) for the blank control group compared to the groups who received 10% NWP, 5% WPH, 10% WPH, 15% WPH, and 0.02% BHA. The results of the 10% WPH and 15% WPH groups were higher and considerably better than those of the 0.02% BHA and blank control groups (*p* < 0.05) after three, five, and seven F–T cycles were completed. Similarly, Su et al. [56] found that the texture score of the fish sausage produced using modified whey protein was greater than that of the control group. It is clear from the above that healthier surimi balls with a reduced loss of sensory quality can be made using the WPH. Moreover, the 15% WPH samples showed increased sensory preference as the F–T duration increased.

## 4. Conclusions

This study examined how different WPH additives affected the quality of surimi balls frozen and thawed several times. In the experiment, 10% NWP, 5% WPH, 10% WPH, 15% WPH, 0.02% BHA, and a blank control were added. Each group’s physical and chemical indices, including its elasticity, hardness, water-holding capacity, cooking loss, carbonyl content, and total sulfhydryl content, were determined. Tukey multiple comparisons were then used to assess the significance of the various WPH addition amounts on surimi balls. The analysis of the aforementioned indicators revealed that fish balls with a 15% WPH addition worked the best.

Studies have shown that the F–T cycle significantly influences the oxidation of fish meat protein and the surimi ball quality. Surimi balls with different WPH concentrations showed considerable variations in their physical properties, protein oxidation, cooking loss, structure, and elasticity during the F–T cycles. The data showed that surimi balls containing 15% WPH, similar to those containing 0.02% BHA, had good oxidation stability and a good sensory quality compared to all other treatments. The results showed that 15% WPH was helpful in keeping protein from oxidizing, which improved the surimi balls’ capacity to hold water. These findings provide a theoretical basis for applying WPH in the food industry and guidelines for generating surimi ball products of the highest standard during cryopreservation.

## Data Availability

Data is contained within the article.
